# Diagnosis and Prognostic Significance of c-Met in Cervical Cancer: A Meta-Analysis

**DOI:** 10.1155/2016/6594016

**Published:** 2016-03-16

**Authors:** Jifeng Peng, Shengnan Qi, Ping Wang, Wanyu Li, Chunxia Liu, Feng Li

**Affiliations:** ^1^Department of Pathology, Shihezi University School of Medicine and The Key Laboratories for Xinjiang Endemic and Ethnic Diseases, Chinese Ministry of Education, Shihezi, Xinjiang 832002, China; ^2^Department of Pathology, The First Affiliated Hospital, Shihezi University School of Medicine, Shihezi, Xinjiang 832002, China

## Abstract

*Objective*. A meta-analysis was conducted to analyze c-Met expression in cervical cancer.* Methods*. Articles related to our study were retrieved from PubMed, Elsevier, and China National Knowledge Infrastructure. State 12.0 was used for literature review, data extraction, and meta-analysis. The random-effects model and fixed-effects model were utilized to pool the relative ratio based on the heterogeneity test in the meta-analysis.* Results.* Nine studies that include data of 685 cervical carcinoma tissues were analyzed. However, three studies did not thoroughly discuss c-Met expression in nonneoplastic cervical tissue; thus, only six studies involving 364 patients and 228 nonneoplastic cervical tissues were included in the review. c-Met expression was higher in cervical cancer (60.99%) than in nonneoplastic cervical tissue (19.74%). Cervical carcinoma, cervical intraepithelial neoplasm, and normal cervical tissue were also examined. Results showed that increasing malignancy resulted in elevated c-Met expression. The relationship between c-Met expression and clinicopathologic features was also evaluated. c-Met expression correlated with disease-free survival, lymph node involvement, and lymphovascular space invasion. No statistical difference was observed between c-Met expression and other clinicopathological factors.* Conclusions.* c-Met is a potential diagnostic and prognostic indicator of cervical cancer.

## 1. Introduction

Cervical cancer, with an estimated death number of 265,700, was the fourth most commonly diagnosed cancer and was the fourth leading cause of cancer death among women worldwide in 2012. Approximately 87% of cervical cancer deaths occurred in developing countries [1–3]. Cervical cancer is also the second leading cancer type in many developing countries, and several mechanisms and factors affect the incidence of this cancer.

c-Met is a hepatocyte growth factor (HGF) receptor and a protooncogene that encodes c-Met gene production. c-Met is also a transmembrane receptor demonstrating independent phosphorylation [4, 5]. HGF and its receptor c-Met play an important role in a series of complex intracellular pathways by specifically binding with each other to regulate tumor invasion, metastasis, and angiogenesis [5–7]. Studies have suggested that HGF/c-Met can stimulate the expression of vascular endothelial growth factor, which can promote endothelial cell division and increase vascular permeability to stimulate angiogenesis while altering tumor matrix and promoting tumor growth and metastasis [8]. c-Met is also overexpressed in human tumors, such as thyroid, gastric, pancreatic, breast, and prostate cancers [9–13].

Moreover, c-Met overexpression is detected in cervical and endometrial cancers [14, 15]. In endometrial cancer, c-Met may exert its effect via the PI3K/Akt pathway, which is dependent on COX-2 upregulation that induces cancer cell resistance to apoptosis [16]. c-Met also plays an important role in the progression of endometrial cancer and is a potential indicator of the effect of hormone disruption [17]. Studies [14, 18] have also detected c-Met expression in cervical cancer. Han et al. [19] used mRNA to detect c-Met expression in 36 cervical cancer and 31 normal cervical tissue cases. They found that the expression of c-Met is higher in cervical cancer than in normal cervical tissue. Refaat et al. [20] detected c-Met overexpression in cervical cancer through immunohistochemistry and revealed that c-Met overexpression is a potential predictive marker and therapeutic target in cervical cancer patients. Walker et al. [21] also found that overexpression of the HGF/c-Met complex strongly correlates with oncogenic HPV (human papillomavirus) and HIV (Human Immunodeficiency Virus) infection in cervical carcinoma. However, these studies involved a small sample size; hence, their data were unconvincing. Most of these studies did not investigate the correlation between c-Met expression and clinical parameters. Moreover, no detailed meta-analysis on the relationship of c-Met expression in cervical cancer with clinical parameters is available. Therefore, the present study performed a meta-analysis on c-Met expression by using published articles on cervical cancer.

## 2. Methods

### 2.1. Publication Search

PubMed, Elsevier, and China National Knowledge Infrastructure were searched using the key words “c-Met,” “Met,” “HGF/c-met,” and “HGF,” with “cervical cancer,” “diagnosis,” and “prognosis.” The most recent research update was published on August 10, 2015. Each article was manually screened. The related articles were read in full text, excluding those that evidently did not comply with the requirements of our meta-analysis.

### 2.2. Inclusion and Exclusion Criteria

The inclusion criteria were as follows: (1) direct immunohistochemical detection of cervical cancer without any restriction on publication year or language; (2) no limitation on age and race; and (3) outcome indicators including clinical parameters, such as age (≥35 years old/<35 years old), histological grade (poor expression versus well and moderate expression), clinical stage (III, IV/I, and II), lymph node metastasis, lymphovascular space invasion (LVSI), parametrial involvement, tumor size, and stromal invasion (≥50% invasion versus 50% invasion), and cancer cell type (squamous carcinoma/nonsquamous carcinoma).

The exclusion criteria were as follows: (1) nondetection of c-Met expression in cervical cancer; (2) no clear positive and negative expression of cervical cancer; and (3) failure to acquire valid data required for relevant clinical parameters, such as age, histological grade, clinical stage, lymph node metastasis, stromal invasion, LVSI, parametrial involvement, tumor size, and cancer cell type.

### 2.3. Data Extraction

Data were carefully and independently extracted from all eligible publications by following the inclusion and exclusion criteria. A study was included in the meta-analysis if investigators have reached a consensus. The data extracted from each study included the name of the first author, year of publication, number of cases, supplier of antibody, clinicopathological parameters, experimental methods, and case collection period.

### 2.4. Statistical Analysis

State 12.0 was used in the meta-analysis. c-Met expression was considered significant when *P* < 0.05. Univariate analysis was performed to examine the differences in c-Met expression between cancer and noncarcinoma tissue. Risk ratios (RRs) or odds ratios (ORs) estimated at 95% confidence intervals (CIs) were used to analyze dichotomous variables. The random-effects model was utilized to calculate the pooled effect. In addition, chi-square test, *I* ^2^ index, and *P* value were used to evaluate the studies. *I* ^2^ index and *P* value were consistent in terms of heterogeneity. The fixed-effects model was employed when *I* ^2^ < 50% and *P* > 0.1, whereas the random-effects model was used when *I* ^2^ > 50% and *P* < 0.1. However, several inconsistencies, such as when *I* ^2^ > 50% and *P* > 0.1, were found between the two models because the *I* ^2^ index takes the degree of freedom into account. As such, *I* ^2^ is recommended as a reference, and the random-effects model was selected. When *I* ^2^ < 50% and *P* < 0.1, *I* ^2^ was recommended as a reference, and the fixed-effects model was selected for statistical analysis.

## 3. Results

### 3.1. Study Selection and Characteristics

**Figure 1:**
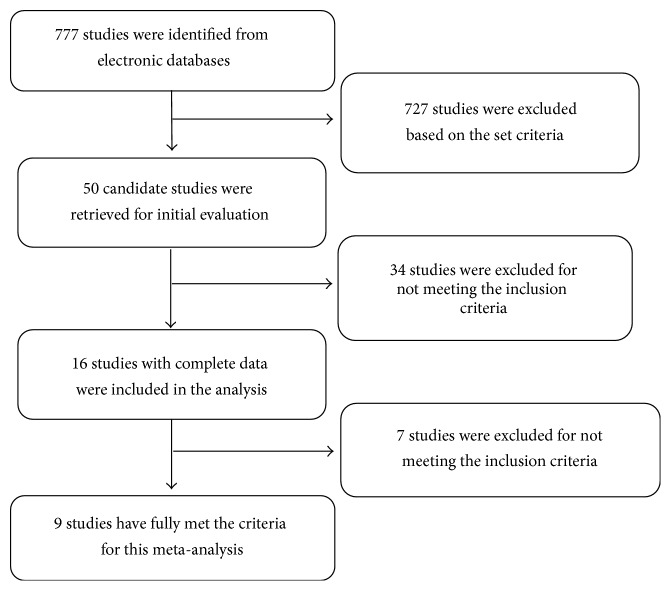
Flow chart of literature review and study selection process.

A total of 727 out of 777 potentially relevant studies were irrelevant to this meta-analysis (Figure 1). The 50 remaining articles were read in detail, and only 16 articles were highly related to our meta-analysis. The full texts of the remaining 16 articles were read. Finally, nine articles [22–30] met all the conditions of this study.

All of the nine articles immunohistochemically detected c-Met protein, and a summary of their basic information is presented in Table 1. Among the nine articles, six thoroughly discussed c-Met expression in cancer and nonneoplastic cervix tissue, whereas three articles did not investigate c-Met expression in nonneoplastic cervix tissue groups. Approximately 364 cervical cancer and 228 nonneoplastic cervix tissues were extracted from the six studies. The c-Met expression in cervical cancer was 60.99% (222/364).

### 3.2. Correlation between c-Met Expression and Tumor Clinical Pathologic Features

**Figure 2:**
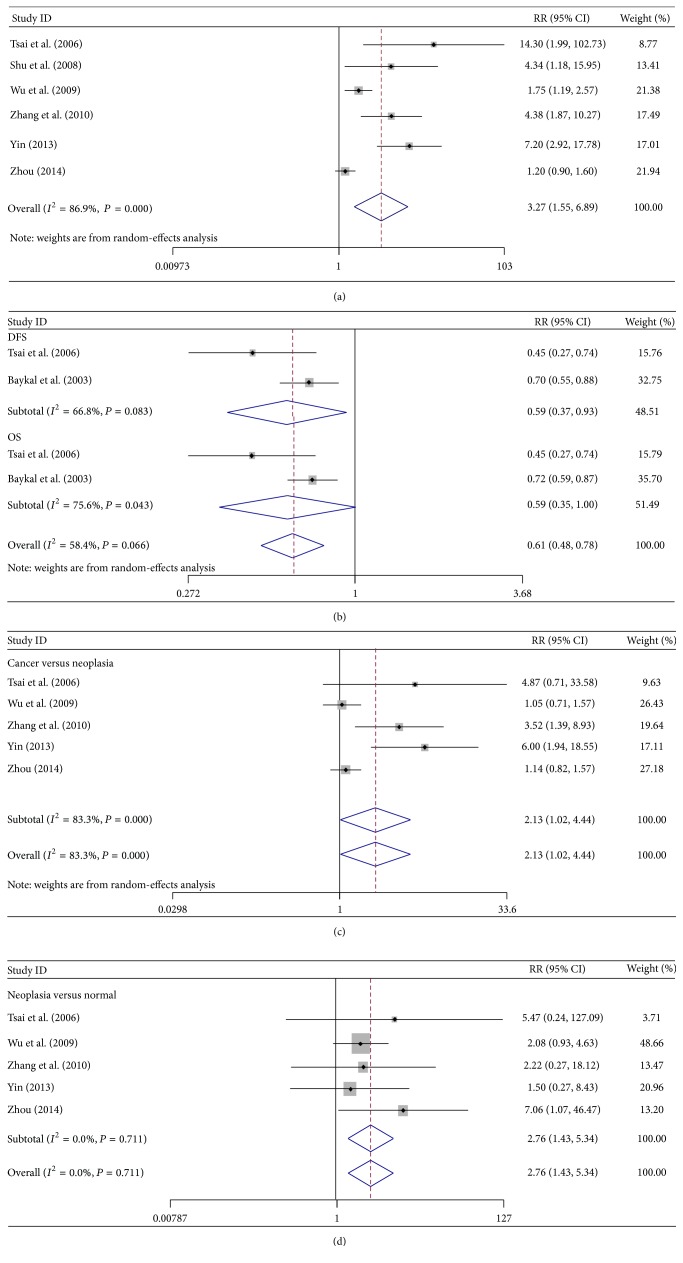
(a) Forest plots of c-Met expression analyses. The random-effects model was used in the analysis. Studies were stratified based on the type of specimen: cervical cancer group versus noncancer group. Squares and horizontal lines correspond to the study-specific RR and 95% CI, respectively. Rhombus represents the summary of RR and 95% CI. (b) Forest plots of c-Met analyses with disease-free survival (DFS) and overall survival (OS). The random-effects model was used. Studies were stratified based on the type of specimen: c-Met expression in cervical cancer and noncancer groups with DFS and OS. (c) and (d) Forest plots of c-Met expression analyses between cervical cancer tissue and intraepithelial neoplasm (c) and between intraepithelial neoplasm and normal cervical tissue groups (d). Weight: the number of cases in each article accounts for the proportion of the total cases. RR: risk ratio. CI: confidence interval (also 95% CI). Red dotted line: invalid line. Blue square: total weight.

A total of 685 cervical cancer patients were analyzed in these nine studies. Only six studies involving 364 cervical cancer patients and 228 nonneoplastic cervix tissue patients were included because three studies provided insufficient data. The c-Met expression was higher in cervical cancer than in nonneoplastic cervix tissue (RR = 3.27; 95% CI: 1.55–6.89, *P* = 0.002). Statistical heterogeneity was also significant (*I* ^2^ = 86.9%, *P* = 0.00001) (Figure 2(a)). The random-effects model was used in the analysis.

Articles that investigated the correlation of c-Met protein expression with disease-free survival (DFS) or overall survival (OS) were examined for this meta-analysis (Table 2). Results showed that c-Met overexpression correlated with poor DFS (RR = 0.59; 95% CI: 0.37–0.93, *P* = 0.025) and demonstrated moderate heterogeneity (*I* ^2^ = 66.8%, *P* = 0.083) (Figure 2(b)). A poor OS tendency was also found in c-Met expression (RR = 0.59; 95% CI: 0.35–1.00, *P* = 0.052), with *P* > 0.05, indicating the absence of a statistical significance.

Invasive cervical cancer, intraepithelial neoplasia (including carcinoma in situ), and nonneoplastic cervical groups were compared in five articles reporting on c-Met expression (Table 2). The cervical cancer group demonstrated a higher c-Met expression (RR = 2.13; 95% CI: 1.02–4.44, *P* = 0.044) and moderate heterogeneity (*I* ^2^ = 83.3%, *P* = 0.0001) compared with the neoplasia group (Figure 2(c)). The result indicates that c-Met expression was higher in the cervical cancer group than in the intraepithelial neoplasia group. c-Met expression was also higher in the intraepithelial neoplasia group than in the normal cervical tissue group (RR = 2.76; 95% CI: 1.43–5.34; *P* = 0.003). The random-effects model was used to detect moderate heterogeneity (*I* ^2^ = 0.0%, *P* = 0.711) (Figure 2(d)).

**Figure 3:**
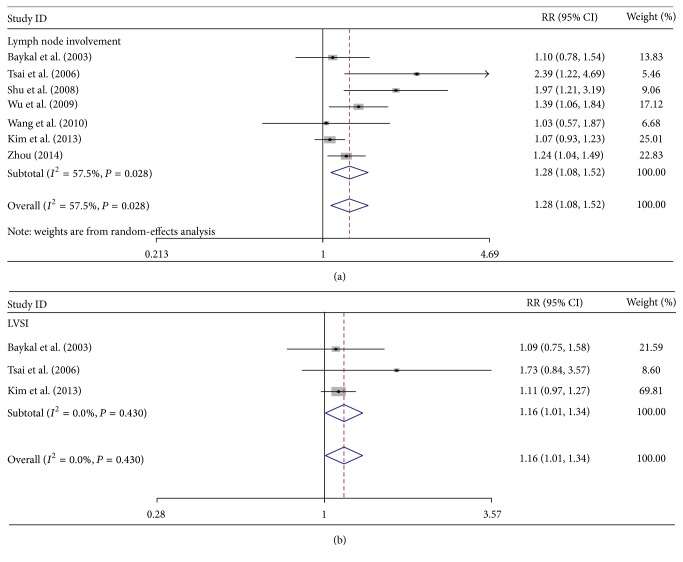
Forest plots of c-Met expression analyses between lymph node metastasis (a) and lymphovascular space invasion (LVSI) (b). Studies were stratified based on the specimen type: lymph node metastasis versus nonlymph node metastasis, and LVSI positive versus LVSI negative.

Clinical parameters, including tumor stage, lymph node involvement, depth of cervical stromal invasion, tumor differentiation (grade), patient age, presence of parametrial involvement, LVSI, tumor size, and cancer cell histologic type, were examined (Table 2). Seven studies assessed the correlation of c-Met expression with lymph node involvement. The pooled RR was 1.28 (95% CI: 1.08–1.52, *Z* = 2.78, *P* = 0.005; Figure 3(a)), indicating that a high c-Met expression involves lymph node metastasis. The random-effects model was used to detect moderate heterogeneity (*I* ^2^ = 57.5%, *P* = 0.028). Moreover, three studies that investigated LVSI were examined. A high c-Met overexpression correlated with a high percentage of LVSI. The pooled RR was 1.16 (95% CI: 1.01–1.34, *Z* = 2.07, *P* = 0.038; Figure 3(b)), and the heterogeneity was low (*I* ^2^ = 0.00%, *P* = 0.430). Clinical stage, deep cervical stromal invasion, histological differentiation, patient age, presence or absence of parametrial involvement, tumor size, and histological cell type were not correlated with c-Met expression (*P* > 0.05).

### 3.3. Publication Bias Analysis

State 12.0 was used to detect publication bias, and most of the points were within the 95% CI, indicating a moderate publication bias.

## 4. Discussion

Cervical cancer is a common gynecological cancer worldwide. Early detection greatly increases the success of patient treatment and prolongs patient survival. Lymph node metastasis and local or regional relapse are the primary causes of death in these patients [31–33]. Therefore, detection of cervical cancer by using reliable biological markers is necessary.

The protooncogene c-Met encodes a growth factor receptor encoding HGF receptor and demonstrates tyrosine kinase activity. c-Met induces the proliferation, movement, and invasion of epithelial cells. Several experimental studies have shown that the activation of HGF/c-Met signal transduction is related to the occurrence and development of human tumors.

c-Met is overexpressed in human tumors, such as thyroid, gastric, pancreatic, breast, and prostate cancers. HGF/c-Met is associated with the occurrence, development, and prognosis of cervical cancer. Manavi et al. [18] used a disposable cervical sampler to obtain six high-risk cervical HPV-positive squamous cells and six normal cervical HPV-negative vaginal squamous cells through cDNA array analysis; they found that c-Met gene cDNA is significantly overexpressed in cervical squamous cell carcinoma. They inferred that the c-Met gene can be used to evaluate the biological behavior and clinical outcome of cervical cancer. Shimabukuro et al. [34] detected HGF/c-Met expression through RT-PCR of the cervical cancer cell line SKG-IIIa, Hela-S3, and cervical cancer mesenchymal cells. In particular, HGF mRNA expression was detected in cervical cancer mesenchymal cells and c-Met mRNA expression in SKG-IIIa and Hela-S3 cells.

c-Met is a receptor tyrosine kinase that plays a vital role in cancer growth by activating mitotic signaling pathways. Interference with c-Met activation may provide an effective approach for cervical cancer treatment [35]. This meta-analysis of nine studies showed that c-Met expression is an important factor for the diagnosis and prognosis of cervical cancer. The use of c-Met as a therapeutic target should be further explored because c-Met is highly expressed in cervical cancer. With further research, the inhibition of c-Met expression can be used as an effective method to treat cervical cancer.

However, the greatest challenge is that only few studies investigated c-Met expression in cervical cancer, and most of the articles that are eligible for meta-analysis reported on data obtained from the same country. Thus, heterogeneity may be observed among data reported in these articles. We did not obtain further clinical follow-up data on c-Met expression in cervical cancer because this factor is relatively a new one. Thus, more robust clinical data are needed to confirm the conclusion of this meta-analysis.

In this meta-analysis, nine articles reporting on c-Met expression in cervical cancer were analyzed. We evaluated the data from nine articles reporting on c-Met expression in 543 cervical cancer and 868 nonneoplastic cervix tissues. The expression of c-Met was significantly lower in nonneoplastic cervical tissue than in cervical cancer. DFS and OS were also examined. The c-Met overexpression in primary tumor correlated with poor DFS. Although c-Met overexpression in primary tumor correlated with OS has no meaning, there was a trend with poor OS about cervical cancer patients. Comparison of clinical parameters showed that cases demonstrating a high c-Met expression in their primary tumors are prone to exhibit lymph node metastasis and LVSI, although this phenomenon is not associated (or correlated) with clinical stage, percentage of cervical stromal invasion, histopathological differentiation, patient age, presence of parametrial involvement, tumor size, and histological cell type. In conclusion, c-Met, as an important factor in tumorigenesis, is a potential diagnostic and prognostic indicator of cervical cancer.

## Figures and Tables

**Table 1:** Characteristics of studies included in the meta-analysis.

First author	Year	Number of patients	Mean age	Stage	Method	Clone number of antibodies	Dilution	c-Met expression (%)	Definition negative	Time to collect cases (year)
Baykal [22]	2003	94	45	I 1B	IHC	Santa Cruz	NR	56/94 (59.6%)	≤25%	1980–1994
Tsai [23]	2006	69	50	I A, I B/II A, and II B	IHC	Santa Cruz	1 : 200	21/69 (30, 4%, ACA)	<10%	1990–2003
Shu [24]	2008	50	NR	I/II	IHC	Zhongshan Beijing Biological Technology Co. Ltd.	NR	31/50 (62.0%)	Nonstaining	2005–2007
Wu [25]	2009	48	49.6	I, II/III, and IV	IHC	Zhongshan Beijing Biological Technology Co. Ltd.	NR	42/48 (87.5%)	Nonstaining	2001–2005
Wang [26]	2010	48	47	II A	IHC	Boster (Wu Han)	NR	35/48 (73.0%)	<1 score	2007-2008
Zhang [27]	2010	71	47	I, II/III, and IV	IHC	Boster (Wu Han)	NR	37/71 (52.1%)	≤1 score	2007.4–2008.2
Kim [28]	2013	179	43.8	I, II/IV	IHC	Abcam	1 : 500	152/179	0 scores	1996–2010
Yin [29]	2013	35	NR	NR	IHC	ZSGB-BIO	NR	18/35 (51.43%)	≤10%	2009.12–2010.8
Zhou [30]	2014	91	43.694 ± 8.714	I/II	IHC	Bioss (Beijing)	1 : 200	73/91 (80.2%)	0%	2010.4–2012.11

Positive-staining cells: staining cell score × positive cell percentage = total score; nonstaining cells: no cell staining; proportion of staining cells: 0, 10%, and 25%; IHC: immunocytochemistry.

**Table 2:** Results of meta-analysis on c-Met expression in cervical cancer.

Outcome of interest	Number of studies	Number of tissue samples	RR/WMD	95% CI	Heterogeneity (%)	*P*	*Z*
Carcinoma and neoplasia	5	E c-Met = 266, C c-Met = 108	2.13	1.02–4.44	*I* ^2^ = 83.3, *P* = 0.0001	0.044	2.01
Neoplasia and normal tissue	5	E c-Met = 108, C c-Met = 105	2.76	1.43–5.34	*I* ^2^ = 0.0, *P* = 0.711	0.003	3.01
DFS	2	E c-Met = 77, C c-Met = 86	0.59	0.37–0.93	*I* ^2^ = 66.8, *P* = 0.083	0.025	2.24
OS	2	E c-Met = 77, C c-Met = 86	0.59	0.35–1.00	*I* ^2^ = 75.6, *P* = 0.043	0.052	1.94
Stage	3	E c-Met = 35, C c-Met = 263	1.33	0.97–1.82	*I* ^2^ = 77.1, *P* = 0.013	0.073	1.79
Cancer cell types	5	E c-Met = 359, C c-Met = 103	1.05	0.81–1.37	*I* ^2^ = 67.8, *P* = 0.015	0.697	0.39
Lymph node involvement	7	E c-Met = 173, C c-Met = 392	1.28	1.08–1.52	*I* ^2^ = 57.5, *P* = 0.028	0.005	2.78
Deep stromal invasion	2	E c-Met = 57, C c-Met = 85	1.31	0.85–2.00	*I* ^2^ = 65.3, *P* = 0.090	0.220	1.23
Differentiation grade	5	E c-Met = 129, C c-Met = 303	1.28	0.98–1.67	*I* ^2^ = 59.2, *P* = 0.044	0.070	1.81
Age	2	E c-Met = 78, C c-Met = 64	0.71	0.50–1.01	*I* ^2^ = 0.0, *P* = 0.480	0.058	1.9
Parametrial involvement	3	E c-Met = 45, C c-Met = 122	2.05	0.93–4.50	*I* ^2^ = 75.3, *P* = 0.017	0.074	1.79
LVSI	3	E c-Met = 122, C c-Met = 204	1.16	1.01–1.34	*I* ^2^ = 0.0, *P* = 0.430	0.038	2.07
Tumor size	2	E c-Met = 56, C c-Met = 86	1.06	0.74–1.51	*I* ^2^ = 54.2, *P* = 0.140	0.758	0.31

C: control group; E: experiment group; RRs: risk ratios; WMD: weighted mean difference; CI: confidence interval; OS: overall survival; DFS: disease-free survival; *I* ^2^: heterogeneity detection; LVSI: lymphovascular space invasion.
